# Combined abdominal and transcranial photobiomodulation is associated with fecal microbiome restructuring: a systems microbiology pre-post case report

**DOI:** 10.3389/fsysb.2026.1920618

**Published:** 2026-09-10

**Authors:** Gabriela N. F. Guimarães, Douglas W. Barrett, F. Gonzalez-Lima

**Affiliations:** 1 Texas Consortium in Behavioral Neuroscience, Department of Psychology, University of Texas at Austin, Austin, TX, United States; 2 Interdisciplinary Neuroscience Program, Division of Pharmacology and Toxicology, and Department of Psychiatry and Behavioral Sciences, University of Texas at Austin, Austin, TX, United States

**Keywords:** 16S rRNA sequencing, case report, fecal microbiome, gut-brain axis, photobiomodulation

## Abstract

Photobiomodulation (PBM) has been proposed as a non-invasive strategy capable of modulating mitochondrial, inflammatory, vascular, and gut-brain axis-related pathways. However, its potential effects on fecal microbiome ecology in neurobehavioral conditions remain poorly characterized. This case report describes paired fecal microbiome findings before and after a 90-day combined abdominal and transcranial PBM protocol in a 6-year-old female with autism spectrum disorder and chronic constipation. PBM was delivered daily using a 1064-nm near-infrared LED device with 54 mW/cm^2^ irradiance, applied for 10 min over the right prefrontal cortex and for 10 min over the infraumbilical and right lower abdomen. Fecal samples collected before and after the intervention were analyzed by 16 S rRNA amplicon sequencing. The baseline sample showed limited taxonomic resolution, with 93.14% assigned to “Other/unclassified,” whereas the post-intervention sample showed a more taxonomically resolved microbial profile, including Firmicutes (53.37%), Bacteroidetes (29.66%), Actinobacteria (7.97%), Verrucomicrobia (4.21%), Euryarchaeota (2.89%), Proteobacteria (1.14%), and a markedly reduced unclassified fraction (0.03%) at the phylum level. At the genus level, the post-intervention profile showed detection or increased representation of taxa commonly discussed in relation to gut microbial ecology, mucosal biology, and short-chain fatty acid metabolism, including Faecalibacterium (7.03%), Bifidobacterium (4.25%), *Bacteroides* (18.82%), Alistipes (5.38%), preserved Akkermansia (3.92%–4.21%), and low-abundance Roseburia. Taxa with context-dependent or uncertain significance, including Sarcina, Collinsella, Parabacteroides, and residual unclassified genera, were also observed and are reported transparently. Caregiver behavioral observations included improved bowel regularity, reduced abdominal distension, more stable mood, reduced irritability and impulsivity, improved communication, and greater social tolerance. The intervention was well tolerated with no reported adverse effects. As a single-patient pre-post case report without sham control, repeated baseline sampling, or functional metagenomic confirmation, these findings cannot establish causality. Nevertheless, they provide a biologically plausible systems-level observation linking combined abdominal and transcranial PBM with a shift in fecal microbiome structure and gastrointestinal-behavioral regulation, supporting future controlled studies integrating longitudinal microbiome profiling, metabolomics, inflammatory biomarkers, autonomic measures, and validated behavioral outcomes.

## 1 Introduction

Photobiomodulation (PBM) refers to the application of non-ionizing red or near-infrared light to modulate cellular function. PBM has been investigated in relation to mitochondrial bioenergetics, inflammatory signaling, tissue repair, vascular regulation, and neuropsychological applications (Cardoso et al., 2021; Rojas and Gonzalez-Lima, 2013). More recently, host-microbiome interactions have been proposed as a potential pathway through which PBM may exert systemic effects. The gut microbiome is increasingly recognized as a central biological interface between the external environment and host physiology. Alterations in microbial diversity, taxonomic composition, and microbial metabolic function have been associated with intestinal barrier integrity, immune tone, systemic inflammation, metabolic regulation, and gut-brain axis signaling (Cryan et al., 2019; Morais et al., 2021). In particular, bacterial taxa associated with short-chain fatty acid metabolism, such as *Faecalibacterium*, *Roseburia*, *Bifidobacterium*, and selected members of the Firmicutes and Actinobacteria phyla, are frequently discussed in relation to intestinal homeostasis and mucosal integrity (Bicknell et al., 2019; Bicknell et al., 2022a).

Initial laboratory and clinical studies suggest that PBM may influence gut microbial ecology. Bicknell et al. reported that abdominal PBM altered gut microbiome composition in healthy mice. Subsequently, Bicknell and Laakso et al. described a human case in which abdominal PBM was associated with increased microbial diversity and changes in genera such as *Akkermansia*, *Faecalibacterium*, and *Roseburia* (Bicknell et al., 2019; Bicknell et al., 2022a; Bicknell et al., 2022b). Their case used repeated fecal sampling before and after PBM and proposed microbiome modulation as a potential mechanism of systemic PBM effects.

Recent mechanistic and controlled clinical evidence supports the selection of the right prefrontal cortex as the transcranial target. Transcranial infrared stimulation at 1064 nm may modulate cerebral energy metabolism and hemodynamics through mitochondrial photoacceptors, particularly cytochrome c oxidase, with placebo-controlled studies reporting effects on cerebral oxygenation, electrophysiological activity, and cognitive performance (Wang et al., 2022). Consistent with this evidence, a randomized sham-controlled trial in adults with ADHD found that a single session of right-prefrontal tPBM increased prefrontal oxygenation and improved inhibitory control compared with sham stimulation (Salehpour et al., 2026). Although these studies did not evaluate pediatric populations, abdominal PBM, or microbiome outcomes, they provide relevant mechanistic and controlled clinical support for the cerebral target used in the present protocol.

Evidence regarding combined abdominal and transcranial PBM remains limited (Gordon et al., 2023; Guimarães et al., 2026; Chen et al., 2021; Sancho-Balsells et al., 2024). This combination is relevant because abdominal PBM may plausibly influence intestinal, immune, vascular, mitochondrial, or enteric nervous system pathways, whereas transcranial PBM may influence cortical, autonomic, and neuroimmune signaling (N. F. Guimarães et al., 2025). The present case report describes paired fecal microbiome findings before and after a combined abdominal and transcranial PBM protocol. The objective of this report is not to establish efficacy, but to describe a biologically plausible and clinically grounded microbiome observation that may guide future studies.

## 2 Case presentation

We report on a 6-year-old female diagnosed with autism spectrum disorder (ASD) level 2 (requiring substantial support) at 4 years old. Early neurobehavioral developmental differences were observed since the first year of life, including delayed gross motor milestones. Her medical history was notable for allergies, seizures, chronic constipation, attention-deficit/hyperactivity disorder (ADHD), and a rare genetic variant involving TUBB2A, which may have contributed to her neurodevelopmental phenotype. At the time of the intervention, she was receiving pharmacotherapy: risperidone (antipsychotic), oxcarbazepine (anticonvulsant), and guanfacine (adrenergic receptor agonist).

The baseline fecal microbiome sample was collected on 12 May 2025, before initiation of the PBM protocol. The combined abdominal and transcranial PBM intervention was started on 14 May 2025 and continued daily for 90 days, ending on 08 August 2025. The post-intervention fecal microbiome sample was collected on 09 August 2025, after completion of the PBM protocol.

During the PBM intervention period, there were no reported changes in diet, supplement use, medications, and no new gastrointestinal intervention was introduced. The patient was not taking any probiotics during the 90-day PBM treatment period. However, gastrointestinal transit and stool characteristics changed during the intervention, with bowel patterns recorded as contextual outcomes rather than controlled variables. Because stool consistency and intestinal transit are themselves associated with fecal microbiome composition, these changes may represent biological mediators, confounders, or both, and their respective contributions cannot be separated in the present design. Accordingly, the microbiome findings were interpreted as a descriptive pre-post biological observation temporally associated with the PBM protocol rather than as evidence of a direct treatment effect. No formal physical examination findings were available beyond caregiver-reported abdominal distension.

Behavioral observations were recorded as contextual outcomes rather than primary efficacy endpoints. A parent-completed measure [Autism Spectrum Quotient (AQ) (Baron-Cohen et al., 2001);] showed a 7.8% decrease in the overall score, from 51 to 47, indicating improvement in autism symptoms after PBM. The greatest improvement was observed in the communication domain, where the deficient subscale score decreased by 50%. Weekly caregiver-reported observations included increased tolerance, calmer mood, improved communication, and longer sleep duration. The patient’s mother also reported that, for the first time, the child was able to sit at the dinner table with her family and ride a bicycle with assistance. In addition, her Applied Behavior Analysis (ABA) therapist reported improvements in task completion and sustained attention.

Given the single-patient pre-post design, no causal inference was assumed. The case was analyzed as an exploratory biological observation, with fecal microbiome composition considered the primary outcome and behavioral changes presented as contextual findings.

## 3 Intervention

The patient received a combined PBM protocol targeting both transcranial and abdominal regions. PBM was administered once daily for 3 months, with sessions performed in the evening to promote relaxation and sleep. Each daily session consisted of 10 min of transcranial PBM followed immediately by 10 min of abdominal PBM.

PBM was delivered using a handheld LED cluster probe equipped with three 1064-nm near-infrared LEDs, with an emission range of 1050–1070 nm. The probe had a total active emission surface area of 23.76 cm^2^, and each LED had a beam divergence of 90°. Each diode emitted 434 mW at full width at half maximum, yielding a total output power of 1.302 W. The probe was separated 7.5 mm from the skin surface by a stopper for both transcranial and abdominal applications. At this working distance, the average irradiance delivered by the three-diode probe was approximately 54 mW/cm^2^. A 10-min exposure therefore delivered an estimated fluence of 32.4 J/cm^2^ and an energy dose of 781 J per modality.

The transcranial application was delivered over the right forehead at the frontopolar point (Fp2 EEG site), targeting the right prefrontal cortex. This brain target was selected because of its relevance to behavioral regulation, executive function, attention, and social-cognitive networks in neuropsychological conditions (Rojas and Gonzalez-Lima, 2013; Wang et al., 2022), and because our previous work demonstrated cognitive and emotional benefits from PBM targeting this region (Barrett and Gonzalez-Lima, 2013; Gonzalez-Lima, 2026; Salehpour et al., 2026). Immediately afterward, abdominal PBM was applied over the infraumbilical region and right lower quadrant, including the area corresponding to McBurney’s point, with the objective of targeting intestinal and gut-brain axis-related pathways (Guimarães et al., 2025).

Each evening session therefore delivered approximately 781 J transcranially and 781 J abdominally, for a total daily radiant energy of approximately 1,562 J. Over the 3-month intervention period, the estimated radiant energy delivered across both modalities was approximately 140.6 kJ. The intervention was well tolerated. Sessions were performed with the patient lying supine, usually in a calm state and often listening to soothing music. No adverse effects were reported during the intervention period.

## 4 Microbiome methods

Fecal microbiome analysis was performed using the Zymo Research 16S Amplicon Sequencing Service. The report indicates that the sequencing workflow included sample receipt, quality evaluation, sample preparation, next-generation sequencing, sequence quality checking, bioinformatic processing, absolute abundance analysis, and data/result delivery.

DNA extraction was performed using a ZymoBIOMICS DNA extraction workflow, and targeted library preparation was conducted using the Quick-16S Plus next-generation sequencing (NGS) Library Prep Kit with primers targeting the V3–V4 hypervariable region of the 16S ribosomal RNA (rRNA) gene. Sequencing was performed on an Illumina NextSeq platform. Bioinformatic processing included DADA2-based inference of unique amplicon sequence variants (ASVs), removal of chimeric sequences, and taxonomic assignment using Uclust within Quantitative Insights Into Microbial Ecology (QIIME) v1.9.1 against the internally curated Zymo Research 16S database. Composition visualization, alpha-diversity, beta-diversity, heatmaps, principal coordinates analysis (PCoA), and related analyses were performed using QIIME and internal scripts.

Given the single-patient, two-timepoint design, the analysis was descriptive. No inferential statistical claims were made.

The 16S rRNA approach was selected pragmatically because it was clinically available, economically feasible, and appropriate for the exploratory taxonomic objective of this paired pre-post case analysis. Both samples were processed using the same laboratory workflow. However, 16S rRNA sequencing does not provide the species- and strain-level taxonomic resolution or functional gene profiling available through shotgun metagenomic sequencing.

## 5 Results

### 5.1 Sequencing and taxonomic classification

Two fecal microbiome samples were included: ASD01. CR, corresponding to the pre-PBM timepoint, and ASD02. CR, corresponding to the post-PBM timepoint.

The pre-intervention sample showed limited taxonomic resolution in the species-level composition output, with 93.14% of the profile assigned to “Other/unclassified.” The remaining classified taxa included *Akkermansia muciniphila* at 3.92%, *Bacteroides xylanisolvens* at 1.14%, *Bacteroides ovatus* at 0.98%, and *Alistipes shahii* at 0.83% (Table 1). In contrast, the post-intervention sample yielded a larger proportion of classifiable taxa. Aggregation of the species-level composition output indicated a restructured phylum-level profile, composed predominantly of Firmicutes (53.37%) and Bacteroidetes (29.66%), with lower relative abundances of Actinobacteria (7.97%), Verrucomicrobia (4.21%), Euryarchaeota (2.89%), and Proteobacteria (1.14%).

**TABLE 1:** Phylum-level fecal microbiome composition before and after combined abdominal and transcranial photobiomodulation.

Phylum	Baseline (Pre-PBM ASD01.CR)	After 90 Days (Post-PBM ASD02.CR)
Other/unclassified	93.14%	0.03%
Firmicutes	0%	53.37%
Bacteroidetes	2.95%	29.66%
Actinobacteria	0%	7.97%
Verrucomicrobia	3.92%	4.21%
Euryarchaeota	0%	2.89%
Proteobacteria	0%	1.14%

Values are expressed as relative abundance (%). PBM, photobiomodulation. A value of 0% indicates that the taxon was not detected or taxonomically classified in the corresponding sample and should not be interpreted as evidence of biological absence.

The marked difference in the proportion of taxonomically classified reads should not be attributed to PBM itself. Taxonomic assignment is a computational outcome influenced by sample quality, DNA extraction and amplification efficiency, sequencing depth and read quality, reference-database coverage, and classifier parameters. Therefore, taxa not detected or classified in the baseline sample cannot be assumed to have been biologically absent.

### 5.2 Genera of biological interest

At the genus level, selected taxa with recognized relevance to gut barrier integrity, short-chain fatty acid production, and microbial metabolic function showed changes after 90 days of combined abdominal and transcranial photobiomodulation (Table 2).

**TABLE 2:** Selected genus-level fecal microbiome findings before and after combined abdominal and transcranial photobiomodulation.

Genus	Baseline (Pre-PBM ASD01.CR)	After 90 Days (Post-PBM ASD02.CR)	Biological relevance
*Akkermansia*	3.92%	4.21%	Mucin-associated genus; often discussed in relation to mucosal barrier biology
*Faecalibacterium*	0%/not detected/classified	7.03%	Butyrate-associated genus
*Bifidobacterium*	0%/not detected/classified	4.25%	Actinobacteria genus commonly associated with gut health
*Roseburia*	0%/not detected/classified	present at low abundance	Butyrate-associated genus
*Bacteroides*	2.12%	18.82%	Major gut genus with diverse metabolic roles
*Alistipes*	0.83%	5.38%	Bacteroidetes-associated genus with context-dependent associations

Values are expressed as relative abundance (%). PBM, photobiomodulation. Non-detection or non-classification does not establish biological absence.

**FIGURE 1:**
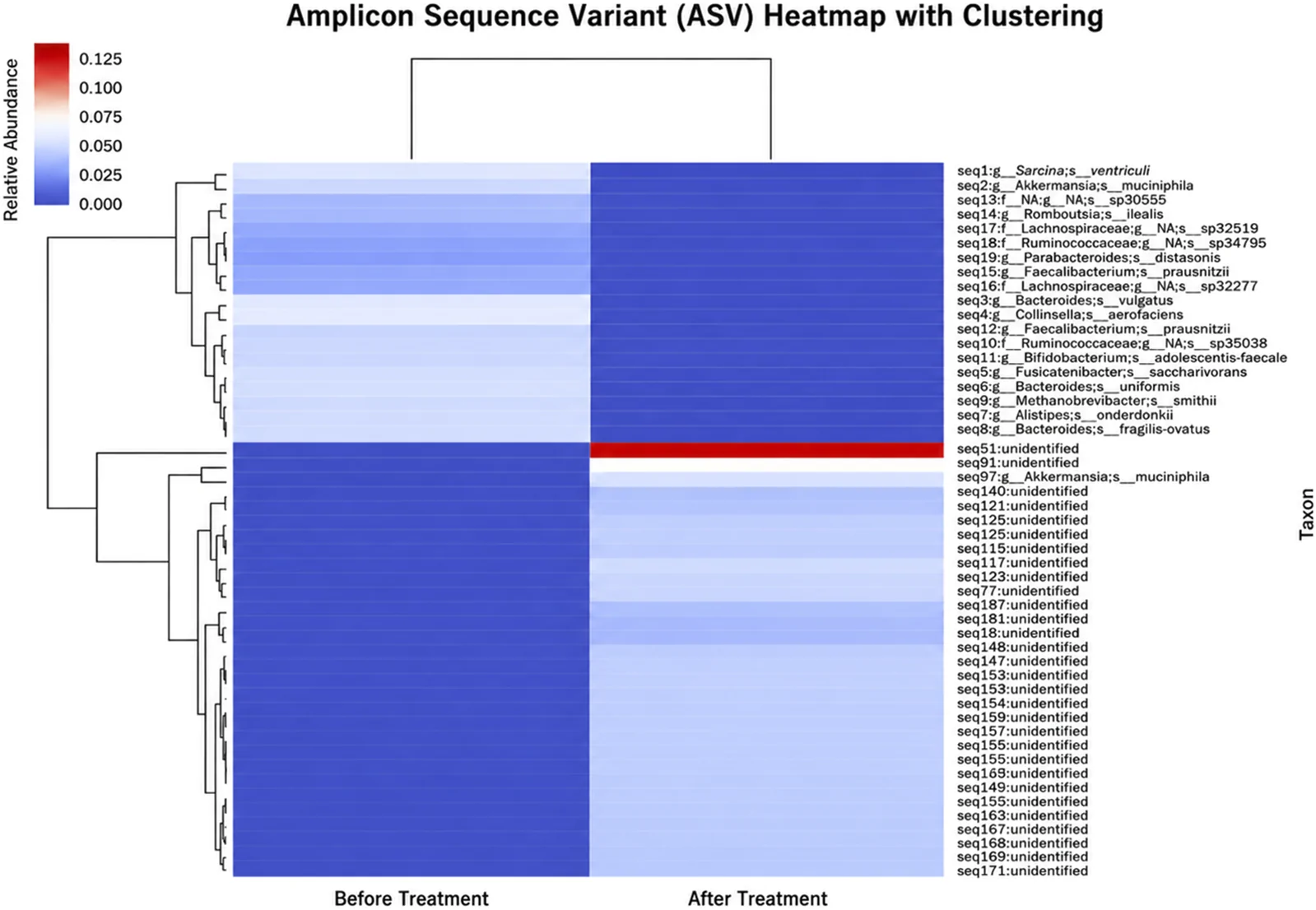
Species-level fecal microbiome heatmap before and after combined abdominal and transcranial photobiomodulation. The color scale represents relative abundance of individual amplicon sequence variants (ASVs), not taxonomic classification confidence. Several ASVs could not be resolved to species level and are labeled as unidentified. The high-abundance unidentified ASV after treatment should therefore be interpreted cautiously and not as evidence of a specific microbial taxon.

The post-intervention sample also included *Sarcina* at 5.80%, *Collinsella* at 3.36%, *Parabacteroides* at 4.41%, and a substantial unclassified genus-level fraction of 22.10%. These findings should be reported rather than omitted, because a credible case report must not selectively describe only favorable taxa (Figure 1).

## 6 Discussion

### 6.1 Comparison with previous PBM-microbiome case literature

This single-patient pre-post case report describes a marked shift in the fecal microbiome taxonomic profile after a 90-day protocol combining abdominal and transcranial PBM. The principal observation was a transition from a baseline profile dominated by unclassified assignments to a post-intervention profile with broader taxonomic resolution and representation of bacterial phyla and genera commonly observed in human gut microbial ecology. At the phylum level, the post-intervention sample showed detectable Firmicutes, Bacteroidetes, Actinobacteria, Verrucomicrobia, Euryarchaeota, and Proteobacteria. At the genus level, the post-intervention sample included *Faecalibacterium*, *Bifidobacterium*, *Akkermansia*, *Bacteroides*, *Alistipes*, and low-abundance *Roseburia*. Although these findings cannot establish causality, they are biologically relevant, because several of these taxa are commonly discussed in relation to mucosal barrier biology, short-chain fatty acid metabolism, and intestinal microbial homeostasis.

The observed post-intervention detection of *Faecalibacterium*, *Bifidobacterium*, *Roseburia*, and preserved *Akkermansia* is directionally relevant to the hypothesis raised by Bicknell et al., who reported increases in beneficial bacteria after PBM, including *Akkermansia*, *Faecalibacterium*, and *Roseburia* (Bicknell et al., 2022a). However, the present case differs substantially, because it includes only two timepoints and combines abdominal and transcranial PBM.

The present observation is directionally consistent with the emerging literature on PBM and microbiome modulation. Bicknell and Laakso et al. reported a human case in which abdominal PBM was followed by increased microbial diversity, increased *Akkermansia*, *Faecalibacterium*, and *Roseburia*, and reduction of selected potentially pathogenic genera (Bicknell et al., 2022a). Importantly, that case included repeated fecal sampling across baseline, pre-PBM, and post-PBM periods, allowing a stronger temporal interpretation than the present two-timepoint design. In contrast, the present case extends the hypothesis into a pediatric neurodevelopmental context and uses a combined abdominal and transcranial PBM protocol. Therefore, the value of this case is not that it replicates the prior design, but that it provides a clinically-grounded observation in a population where gut-brain axis dysfunction, gastrointestinal symptoms, immune signaling, and behavioral regulation may be biologically intertwined.

### 6.2 Mechanistic interpretation

A mechanistic interpretation should remain conservative but is plausible. Abdominal PBM may influence the intestinal environment through host-mediated mechanisms rather than by directly irradiating luminal bacteria. In our recent systematic review of abdominal PBM and the gut-brain axis, the most consistent mechanistic themes included mitochondrial bioenergetic stimulation, modulation of inflammatory pathways, vagal or autonomic signaling, regulation of gut-derived metabolites, and shifts in microbial diversity (Guimarães et al., 2025). These mechanisms provide a coherent framework for interpreting the present case. PBM may improve epithelial mitochondrial function, redox balance, mucosal immune tone, and microvascular perfusion. These host-level changes could alter the ecological conditions that shape microbial composition. In this model, the microbiome is not necessarily a direct photonic target, but rather a downstream biological readout of changes in the intestinal niche (Guimarães et al., 2025). A putative literature-informed functional interpretation of these microbial shifts is warranted, although direct functional inference is limited by the use of 16 S rRNA sequencing without shotgun metagenomics, metabolomics, or short-chain fatty acid quantification.

From a functional perspective, the post-intervention or increased detection of genera such as Faecalibacterium, Bifidobacterium, and Roseburia after PBM is relevant, because these taxa are commonly associated with short-chain fatty acid metabolism, microbial cross-feeding, and intestinal homeostasis (Tamanai-Shacoori et al., 2017; Miquel et al., 2013; Rivière et al., 2016). Faecalibacterium and Roseburia are frequently discussed as butyrate-associated genera, whereas Bifidobacterium may contribute to acetate and lactate production and support cross-feeding interactions that favor butyrate-associated taxa (Miquel et al., 2013; Tamanai-Shacoori et al., 2017). Akkermansia, which was present at baseline and remained detectable after the intervention, is a mucin-associated genus often linked to mucus-layer ecology and mucosal barrier biology (De Vos, 2017). The post-intervention increase in *Bacteroides* may suggest broader polysaccharide fermentation capacity and metabolic flexibility within the intestinal ecosystem, while changes in Alistipes should be interpreted cautiously, because of its context-dependent associations with host diet, inflammatory tone, bile acid metabolism, and microbial community structure (Parker et al., 2020). Similarly, the detection of Sarcina, Collinsella, Parabacteroides, and a substantial residual unclassified genus-level fraction reinforces that the post-intervention microbiome should not be interpreted as uniformly beneficial (Chen et al., 2016). Rather, these findings suggest a shift toward a more taxonomically resolved and potentially more metabolically diverse microbial community, with putative relevance to short-chain fatty acid metabolism, mucin-associated ecology, carbohydrate fermentation, and host-microbiome metabolic cross-talk (Zafar and Saier, 2021; Rivière et al., 2016; Kaoutari et al., 2013).

### 6.3 Behavioral observations

The behavioral observations in this case should be considered contextual rather than primary efficacy outcomes. The parent-completed AQ measure showed a modest reduction in the overall score, with the largest change observed in the communication domain. Caregiver-reported observations described a more stable mood, greater overall happiness, improved willingness to engage in activities and try new experiences, better communication through additional sounds, sound combinations, words, and signs, and improved social interaction with family members, including more tolerant play with her sister. The caregiver also reported reductions in aggression and impulsivity, although difficult days persisted and appeared to be influenced by bowel function, hunger, or general discomfort. Notably, one of the most consistent caregiver-observed changes was improved bowel regularity, with daily bowel movements during the PBM intervention period, despite prior chronic constipation requiring daily polyethylene glycol. This improvement was associated with a visibly less distended abdomen and better mood regulation. The caregiver reported that changes became more consistent after approximately 5–6 weeks, suggesting that longer intervention periods, such as 12 weeks, may be more informative than shorter protocols for evaluating stable behavioral and gastrointestinal changes.

These observations are clinically meaningful but cannot be attributed specifically to PBM or to microbiome changes. Rather, they support the rationale for future studies integrating behavioral outcomes with gastrointestinal symptoms, stool patterns, microbiome profiling, metabolomics, inflammatory markers, autonomic measures, and validated caregiver-reported scales. Taken together, these findings support a systems-level hypothesis in which PBM may influence the intestinal ecosystem indirectly through host-mediated changes in mitochondrial function, mucosal immune tone, vascular perfusion, and epithelial barrier biology, with the fecal microbiome serving as a downstream ecological readout rather than an isolated therapeutic target.

## 7 Limitations

Several limitations are central to the interpretation of this case. First, this is a single-patient pre-post case report without sham control, untreated comparison, or repeated baseline sampling or technical replicates. Consequently, the findings cannot distinguish an intervention-associated effect from normal within-person variability, temporal fluctuation, or analytical variation. The absence of intermediate and post-treatment samples also precludes determination of when the observed difference emerged, whether it developed gradually or transiently, whether it remained stable after treatment, and how it related temporally to the gastrointestinal and behavioral observations.

Second, the baseline sample had a very high “Other/unclassified” fraction. This may reflect biological composition, sample quality, DNA extraction or amplification efficiency, sequencing depth and read quality, database coverage, classifier performance or other technical factors. Because no technical replicate, re-extraction, or independent reanalysis of the raw sequence data was performed, differential analytical or bioinformatic bias between samples cannot be excluded. The post-intervention reduction in the unclassified fraction may therefore reflect a true biological difference, a technical difference, or a combination of both. Accordingly, the greater taxonomic resolution of the post-intervention sample should not be interpreted as evidence of microbiome improvement or attributed directly to PBM. The findings are described as differences in detection and taxonomic assignment rather than definitive appearance or disappearance of individual microbial populations, and taxa not detected or classified at baseline cannot be assumed to have been biologically absent.

Third, 16 S rRNA sequencing provides limited taxonomic information and does not directly establish microbial function, metabolite production, host inflammatory activity, or epithelial barrier status. Compared with shotgun metagenomics, it provides less reliable species- and strain-level identification and does not directly characterize microbial genes or metabolic pathways.

Fourth, because the protocol combined abdominal and transcranial PBM, the relative contribution of each modality cannot be separated. Abdominal PBM may have influenced intestinal and immune pathways, while transcranial PBM may have contributed to behavioral regulation, autonomic tone, sleep, or neuroimmune signaling.

Despite these limitations, this case is relevant because it aligns with a broader translational hypothesis: PBM may exert systemic effects through the interaction between mitochondrial metabolism, mucosal immunity, microbial ecology, and gut-brain signaling. The present case should therefore be framed as hypothesis-generating. Its strongest contribution is not proof of efficacy, but the documentation of an observed, biologically plausible microbiome shift after a clearly described combined abdominal and transcranial PBM protocol in a child with ASD. Future studies should include repeated longitudinal stool sampling, systematic documentation of diet and medications, sham comparison, shotgun metagenomics, absolute abundance analysis, short-chain fatty acid quantification, inflammatory markers, metabolomics, autonomic measures, and validated behavioral scales to determine whether PBM-associated microbiome changes are reproducible, stable, and clinically meaningful.

## 8 Conclusion

This pre-post case report describes fecal microbiome changes after combined abdominal and transcranial PBM. The post-intervention sample showed a more taxonomically-resolved microbial profile and included several genera associated with gut ecological function, including *Faecalibacterium*, *Bifidobacterium*, *Akkermansia*, *Bacteroides*, and *Roseburia*. These findings are exploratory and cannot establish causality. Future studies should use repeated sampling, include appropriate controls, track diet and medications, and incorporate microbiome, metabolomic, inflammatory, and functional analyses.

## Data Availability

The raw 16S rRNA amplicon sequencing data generated and analyzed in this study have been deposited in the NCBI Sequence Read Archive (SRA) under BioProject accession number PRJNA1521790. The data are publicly available at https://www.ncbi.nlm.nih.gov/bioproject/PRJNA1521790.

## References

[B1] Baron-Cohen S. Wheelwright S. Skinner R. Martin J. Clubley E. (2001). The autism-spectrum quotient (AQ): evidence from asperger Syndrome/high-functioning autism, males and females, scientists and mathematicians. J. of Autism Dev. Disord. 31 (1), 5–17. 10.1023/A:1005653411471 11439754

[B2] Barrett D. W. Gonzalez-Lima F. (2013). Transcranial infrared laser stimulation produces beneficial cognitive and emotional effects in humans. Neuroscience 230 (January), 13–23. 10.1016/j.neuroscience.2012.11.016 23200785

[B3] Bicknell B. Liebert A. Johnstone D. Kiat H. (2019). Photobiomodulation of the microbiome: implications for metabolic and inflammatory diseases. Lasers in Med. Sci. 34 (2), 317–327. 10.1007/s10103-018-2594-6 30074108

[B4] Bicknell B. Laakso E.-L. Liebert A. Kiat H. (2022a). Modifying the microbiome as a potential mechanism of photobiomodulation: a case report. Photobiomodulation, Photomed. Laser Surg. 40 (2), 88–97. 10.1089/photob.2021.0057 34962422

[B5] Bicknell B. Liebert A. McLachlan C. S. Kiat H. (2022b). Microbiome changes in humans with parkinson’s disease after photobiomodulation therapy: a retrospective study. J. Personalized Med. 12 (1), 49. 10.3390/jpm12010049 35055364 PMC8778696

[B6] Cardoso F. D. S. Gonzalez-Lima F. Silva S. G.Da (2021). Photobiomodulation for the aging brain. Ageing Res. Rev. 70 (September), 101415. 10.1016/j.arr.2021.101415 34325071

[B7] Chen J. Wright K. Davis J. M. Jeraldo P. Marietta E. V. Murray J. (2016). An expansion of rare lineage intestinal microbes characterizes rheumatoid arthritis. Genome Med. 8 (1), 43. 10.1186/s13073-016-0299-7 27102666 PMC4840970

[B8] Chen Q. Wu J. Dong X. Yin H. Shi X. Su S. (2021). Gut flora-targeted photobiomodulation therapy improves senile dementia in an Aß-Induced alzheimer’s disease animal model. J. Photochem. Photobiol. B Biol. 216 (March), 112152. 10.1016/j.jphotobiol.2021.112152 33610085

[B9] Cryan J. F. O’Riordan K. J. Cowan C. S. M. Sandhu K. V. Bastiaanssen T. F. S. Boehme M. (2019). The microbiota-gut-brain axis. Physiol. Rev. 99 (4), 1877–2013. 10.1152/physrev.00018.2018 31460832

[B10] De Vos W. M. (2017). Microbe profile: akkermansia muciniphila: a conserved intestinal symbiont that acts as the gatekeeper of our mucosa: this article is part of the microbe profiles collection. Microbiology 163 (5), 646–648. 10.1099/mic.0.000444 28530168

[B11] Gonzalez-Lima F. (2026). “Cognitive enhancement through transcranial infrared laser stimulation: insights from our research,” in International Review of Neurobiology (Elsevier). 10.1016/bs.irn.2026.01.015 42613142

[B12] Gordon L. C. Martin K. L. Torres N. Benabid A. Mitrofanis J. Stone J. (2023). Remote photobiomodulation targeted at the abdomen or legs provides effective neuroprotection against parkinsonian MPTP insult. Eur. J. Neurosci. 57 (9), 1611–1624. 10.1111/ejn.15973 36949610 PMC10947039

[B13] Guimarães N. F. G. Dos Santos Cardoso F. Gamboa L. Barrett D. W. Gonzalez-Lima F. (2025). Abdominal photobiomodulation and the gut-brain axis: a systematic review of mechanistic and translational evidence. Biomedicines 13 (12), 3042. 10.3390/biomedicines13123042 41463053 PMC12730906

[B14] Guimarães G. N. F. Salehpour F. Schwartz J. Barrett D. W. Gonzalez-Lima F. (2026). Abdominal and transcranial photobiomodulation as a gut–brain axis therapy in Down syndrome regression disorder: a translational case report. Clin. Transl. Neurosci. 10 (1), 1. 10.3390/ctn10010001

[B15] Kaoutari A.El Armougom F. Gordon J. I. Raoult D. Henrissat B. (2013). The abundance and variety of carbohydrate-active enzymes in the human gut microbiota. Nat. Rev. Microbiol. 11 (7), 497–504. 10.1038/nrmicro3050 23748339

[B16] Miquel S. Martín R. Rossi O. Bermúdez-Humarán L. G. Chatel J. M. Sokol H. (2013). Faecalibacterium prausnitzii and human intestinal health. Curr. Opin. in Microbiol. 16 (3), 255–261. 10.1016/j.mib.2013.06.003 23831042

[B17] Morais L. H. Schreiber H. L. Mazmanian S. K. (2021). The gut microbiota–brain axis in behaviour and brain disorders. Nat. Rev. Microbiol. 19 (4), 241–255. 10.1038/s41579-020-00460-0 33093662

[B18] Parker B. J. Wearsch P. A. Veloo A. C. M. Rodriguez-Palacios A. (2020). The genus alistipes: gut bacteria with emerging implications to inflammation, cancer, and mental health. Front. in Immunol. 11 (June), 906. 10.3389/fimmu.2020.00906 32582143 PMC7296073

[B19] Rivière A. Selak M. Lantin D. Leroy F. Vuyst L.De (2016). Bifidobacteria and butyrate-producing Colon bacteria: importance and strategies for their stimulation in the human gut. Front. in Microbiol. 7 (June), 979. 10.3389/fmicb.2016.00979 27446020 PMC4923077

[B20] Rojas J. C. Gonzalez-Lima F. (2013). Neurological and psychological applications of transcranial lasers and LEDs. Biochem. Pharmacol. 86 (4), 447–457. 10.1016/j.bcp.2013.06.012 23806754

[B21] Salehpour F. Barrett D. W. Mirji A. Farzamnia A. Burugu V. Shah N. (2026). Transcranial photobiomodulation improves prefrontal oxygenation and impulse control in adults with ADHD: a randomized controlled trial. BMC Psychiatry. 10.1186/s12888-026-08391-5 42449279 PMC13651166

[B22] Sancho-Balsells A. Borràs-Pernas S. Flotta F. Chen W. Del Toro D. Rodríguez M. J. (2024). Brain–gut photobiomodulation restores cognitive alterations in chronically stressed mice through the regulation of Sirt1 and neuroinflammation. J. Affect. Disord. 354 (June), 574–588. 10.1016/j.jad.2024.03.075 38490587

[B23] Tamanai-Shacoori Z. Smida I. Bousarghin L. Loreal O. Meuric V. Fong S. B. (2017). *Roseburia* spp.: a marker of health? Future Microbiol. 12 (2), 157–170. 10.2217/fmb-2016-0130 28139139

[B24] Wang X. Gonzalez-Lima F. Liu H. (2022). “Transcranial infrared laser stimulation,” in The Oxford Handbook of Transcranial Stimulation. Editors Wassermann E. M. Peterchev A. V. Ziemann U. Lisanby S. H. Siebner H. R. Walsh V. 2nd ed. (Oxford University Press). 10.1093/oxfordhb/9780198832256.013.10

[B25] Zafar H. Saier M. H. (2021). Gut *bacteroides* species in health and disease. Gut Microbes 13 (1), 1848158. 10.1080/19490976.2020.1848158 33535896 PMC7872030

